# A new species of *Amblyodus* Westwood, 1878 (Coleoptera, Melolonthidae, Dynastinae) from South America

**DOI:** 10.3897/zookeys.75.884

**Published:** 2011-01-12

**Authors:** Paschoal Coelho Grossi, Everardo José Grossi

**Affiliations:** 1 Universidade Federal do Paraná, Centro Politécnico, Departamento de Zoologia, Caixa Postal 19007, CEP 81531-980, Curitiba, Paraná, Brazil; 2 Caixa Postal 96887, CEP 28610-974, Nova Friburgo, Rio de Janeiro, Brazil

**Keywords:** Amazonian subregion, Phileurini, South America, taxonomy

## Abstract

A second species of Amblyodus Westwood, 1878, Amblyodus castroi **sp. n.**, is described from the northern South America based on 12 specimens from Brazil and Peru (Amazonian subregion). The new species is here compared with the type species of the genus, Amblyodus taurus Westwood, 1878 and both species and their male genitalia are illustrated. Diagnostic characters for the genus are discussed, especially the metatibial teeth. A distribution map including the type species and the new species is provided. The genus Amblyodus is recorded for the first time from Peru and from Brazil states of Pará and Rondônia.

## Introduction

Amblyodus was described by Westwood (1878) and until now was known only by the type species, Amblyodus taurus Westwood, restricted to some Central American countries (Blackwelder 1944; Endrödi 1977, 1985; Lachaume 1992; Ratcliffe 2003 and Ratcliffe and Cave 2006). In a recently published paper, Gasca and Aguiar (2008) recorded the genus for the first time from South America (Brazilian Amazon) based on six specimens collected between the Juruá and Purus rivers. However, they wrongly identified the species as Amblyodus taurus.

After studying these specimens and additional six specimens, three from Peru and three from Brazil, we concluded that all these material belong to a new species of Amblyodus which is here described and compared with the type species of the genus. The diagnostic characters are discussed. A distribution map is provided, including a new country locality from Peru.

## Material and methods

The specimens were examined under a dissecting stereomicroscope at magnification of 0.8–50×. Description of external morphological features and conventions follow in part Endrödi (1985) and Ratcliffe and Cave (2006). Images of specimens were captured with a digital camera and assembled in the Automontage software. Label data were separated by single slash “/” for the type material.

The phylogenetic species concept of Wheeler and Platnick (2000) was used in this work: “A species is the smallest aggregation of (sexual) populations or (asexual) lineages diagnosable by a unique combination of characters states.”

Specimens examined for this work were provided by seven institutions and private collections. Acronyms for institutions are mostly taken from Samuelson et al. (2001), as follows:

CEMTColeção Entomológica da Universidade Federal do Mato Grosso, Cuiabá, MT, Brazil (F. Z. Vaz-de-Mello)

EPGCEverardo and Paschoal Grossi Private Collection, Nova Friburgo, RJ, Brazil (E. J. Grossi)

JSCJochen P. Saltin Private Collection, Niedernhausen/Taunus, Germany (J. P. Saltin)

IOCInstituto Oswaldo Cruz, Rio de Janeiro, RJ, Brazil (J. M. Costa)

MIISMuseo Insectarium Internacional de Santiago, Sanriago, Chile (S. Castro)

UFAMUniversidade Federal do Amazonas, Manaus, AM, Brazil (N. O. Aguiar)

MIUPMuseo de Invertebrados G. B. Fairchild, Universidade de Panamá, Panamá (R. A. Cambra T.)

### Key to the Amblyodus species

**Table:** 

1	Frontal horns distinctly divergent. Pronotal disc weakly declivous, almost horizontal, punctures predominantly C-shaped; lateral carina not high. Internal margin of parameres almost parallel; apical external carina of parameres in lateral view complete	Amblyodus taurus Westwood
–	Frontal horns not distinctly divergent, instead parallel. Pronotal disc more declivous, with punctures predominantly reticulated, not C-shaped; lateral carina high. Internal margin of parameres concave; apical external carina of parameres in lateral view incomplete	Amblyodus castroi sp. n.

## Taxonomy

### Amblyodus castroi

Grossi & Grossi sp. n.

urn:lsid:zoobank.org:act:37121595-32A5-4339-93DD-BC7F1AE006B9

Figs 1–8 13, 14 

#### Material examined.

Holotype male, dissected, labeled: a) “male symbol label”; b) “Brasil, Amazonas, Uarini,/ 03°02'57"S, 65°41'42"W,/ 22/VII-03/VIII/1995, P./ Bührnhein, N.Aguiar & al.”; c) handwritten label “NO ALBURNO DE/ TRONCO CAÍDO/ 01/VIII/1995” (UFAM).

**Paratypes**, 4 males and 7 females labeled: 1 female a) “female symbol label”, b) “Brasil, Amazonas, Uarini,/ 03°02'57"S, 65°41'42"W,/ 22/VII-03/VIII/1995, P./ Bührnhein, N.Aguiar & al.”; c) handwritten label “NO ALBURNO DE/ TRONCO CAÍDO/ 01/VIII/1995” (CEMT). 3 females labeled “Brasil, Amazonas, Coari,/ Duto Urucu/Porto Terminal,/ 04°50'16"S, 65°20'36"W,/ 16/VI/1996, Buhmheim (*sic*),/ PF, Aguiar, NO, Arruda,/ AMR & Gualberto, TL col.” (UFAM). 1 male labeled a) “Brasil, Amazonas, Coari,/ Duto Urucu/Porto Terminal,/ 04°50'16"S, 65°20'36"W,/ 16/VI/1996, Bühmheim (*sic*),/ P.F. & Aguiar, N.O. col.”; b) handwritten label “Em tronco/ caído” (EPGC). 1 female labeled “PERU, Junin, Puerto/ Ocopa, I-2007, 600m/ 11°07'50"S, 74°17'46"W/ S.Castro col.” (EPGC). 1 female a) “BRASIL: Rondônia: Porto/ Velho, margem direita/ do Rio Madeira, IX-2004” (EPCG). 1 male labeled “PERU, Ucayali, Via/ Puerto Ocopa-Ucayali,/ XII-2009, 1200m,/ 11°03'51"S, 74°15'39"W/ S. Castro col.” (MIIS); 1 male labeled “PERU, Junin,Huerto/ Eden c.a, near Puerto/ Prado XII-2009/ 1800m, local collector” (JSC). 1 male labeled a) “BR 14, Km 92/ Pará, Brasil/ E. Lobato XII. 60” (IOC). 1 female labeled a) handwritten label “S. Paulo de/ Olivença/ Solimões/ VIII. – Fassl”, b) “Coleção/ J. F. Zikan” (IOC). All paratypes additionally labeled, yellow label, “Amblyodus castroi/ PARATYPE/ Grossi & Grossi.

#### Diagnosis.

Head with frontal horns less divergent, being almost parallel (Fig 3). Pronotal disc more declivous than in Amblyodus taurus with lateral carinae distinctly higher in males majors (Fig 4), posterior furrow deeply excavated as opposed to a less excavated furrow in Amblyodus taurus (Figs 9 and 10). Aedeagus with internal margin of parameres concave, not parallel; external margin less constricted than in Amblyodus taurus where internal margin is almost parallel, and external margin abruptly constricted (Figs 11 and 12).

#### Holotype description

**Figures 1–2.**
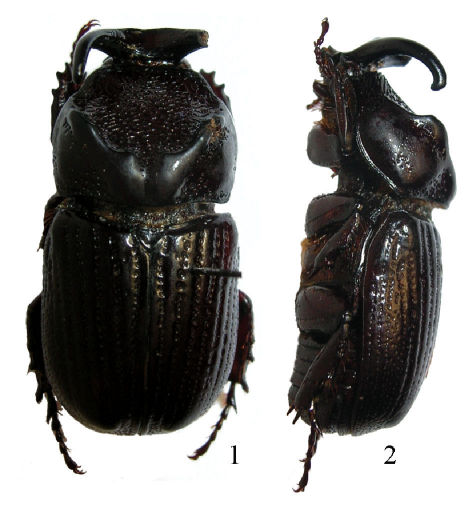
Habitus of Amblyodus castroi sp. n., male holotype. **1** Dorsal view **2** Lateral view.

(Figs 1 and 2). Total body length 20 mm. Total pronotal width 9.2 mm. Body elongated, about 2 times longer than wide, color dark brown dorsally and reddish brown ventrally; dorsal surface glabrous, with sterna being setose. *Head*: Surface smooth and glabrous with sparse micropunctures; in frontal view with two lateral, concave areas with scattered C-shaped puntures. Clypeus subtriangular, laterally bordered and slightly upturned, convex and with acute apex. Canthi intruding upon eyes just before first third. Frons with two erect, diverging, posteriorly recurving horns. Interocular width equals 3.3 mm. Mandibles tridentate with inner tooth more acute; teeth upturned. Antennae 10 segmented with scape as long as the 6 next segments; club with segments subequal in length. *Pronotum:* Shape trapezoidal, narrower than both elytra together; discal area with C-shaped punctures combined with reticulated areas, flat and declivous; anterior border incomplete. Lateral and posterior sides of pronotal depression strongly carinated, anterior carina more pronounced, higher, being convex and wider posteriorly; carina at base weakly emarginate; carina posteriorly interrupted at middle by a longitudinal furrow, internally weakly angulate; furrow about 4.5 times narrower than head width. Anterior angles acute, posterior angles rounded. Outer side and lateral posterior side of pronotal surface punctate; punctures ocellate, sometimes coalescent, posteriorly larger. *Elytra:* Discal area with 5 rows of large ocellate punctures, rows weakly impressed. Interstriae slightly convex, with scattered small punctures. Lateral edges with 5 similar rows of ocellate punctures, instead of by some ocellate punctures between third and fourth rows. *Pygidium:* Shape convex in lateral view; surface densely punctate; punctures ocellate, moderately sized, elongate near dorsal margin. Sixth ventrite sinuous at middle. *Legs:* Protibiae quadridentate, proximal tooth smaller, distal ones bigger. Metatibiae with 5 apical teeth. *Parameres:* Shape symmetrical, narrower at apex; apex dilated, laterally roundly angulated, apically rounded, triangle shaped; internal side of each paramera concave, not parallel (Fig 5). In lateral view shaft constricted, with a post-anterior concavity; dorso-ventral carina incomplete, not reaching ventral margin; with a flat, truncate tooth near base (Fig 6).

Female paratypes. Similar to male in general aspects except for the following characters: *Head:* Frontal horns less developed and never reaching pronotal middle. *Pronotum:* Pronotal depression narrower, near anterior margin smooth, not punctate; lateral carina smaller with a weak acute projection before first third (Figs 7 and 8); pronotal furrow longer, ending just before posterior margin. *Pygidium:* Shape wider laterally and shorter, with punctures smaller. Sixth ventrite not sinuous at middle.

**Figures 3–14.**
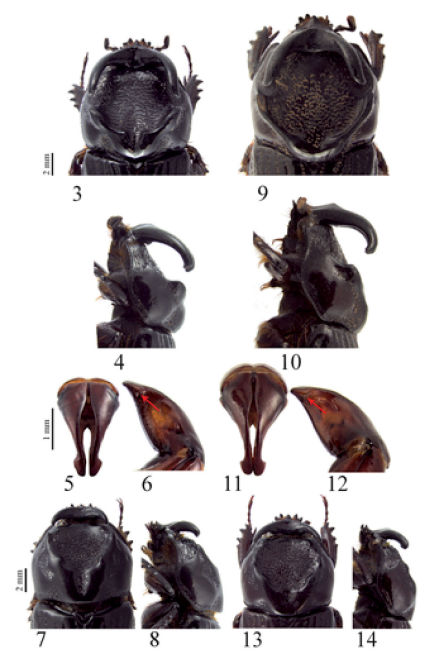
Amblyodus spp. **3–4** Head and pronotum of a male major of Amblyodus castroi in dorsal and lateral views respectively **5–6** Parameres of Amblyodus castroi in caudal and lateral views respectively **7–8** Head and pronotum of a female of Amblyodus castroi in dorsal and lateral views respectively **9–10** Head and pronotum of Amblyodus taurus in dorsal and lateral views respectively **11–12** Parameres of Amblyodus taurus in caudal and lateral views respectively **13–14** Head and pronotum of a male minor of Amblyodus castroi in dorsal and lateral views respectively. Arrows indicate apical lateral carina, **6** (incomplete) and **12** (complete).

#### Variation of male paratypes.

Smaller males possess less developed and less posteriorly recurving frontal horns. Pronotal carina very weakly pronounced (Figs 13 and 14).

#### Etymology. 

The species is named *castroi* in homage of Sergio Castro who provided us with part of the paratypes from Peru.

#### Remarks.

When recording the new occurrence of Amblyodus taurus for South America, Gasca and Aguiar (2008) misapplied the name Amblyodus taurus to the material they had on hand. Actually both species of Amblyodus are similar in appearance, which is common in Phileurini. The authors also stated that the only two genera of the tribe that possess elongate frontal horns were Amblyodus and Oryctophileurus Kolbe, 1910. However, Ceratophileurus Ohaus, 1911 also possess this character in both sexes (Gillett et al. 2010).

Amblyodus and Oryctophileurus share the lateral pronotal carina and pronotal shape: elongate, widely flattened, depressed with discal depression totally wrinkled. Additionally, both genera have almost no external sexual dimorphism, as females also possess a well-developed frontal horn, a feature that makes the distinction between genders difficult, being the shape of the last sternite the better way to determine gender in these cases. Moreover parameres have almost the same shape, and the shape of prosternal process is also shared between Amblyodus and Oryctophileurus, namely flat ventrally and with a spine-like process posteriorly. Based on several morphological characters, there is a possibility that Oryctophileurus could be a synonym of Amblyodus because the unique feature to distinguishing both genera is the number of frontal horns, one or two, which is not a good character within Dynastinae as some genera are usually extremely allometric. Only with the examination of the type species of Oryctophileurus, will it be possible to affirm if they are congeneric or not.

The holotype male is an incomplete specimen and it was apparently damaged by an axe during collecting inside dead logs. It is the same male specimen illustrated by Gasca and Aguiar (2008, figure 1, male in lateral view).

**Figure 15.**
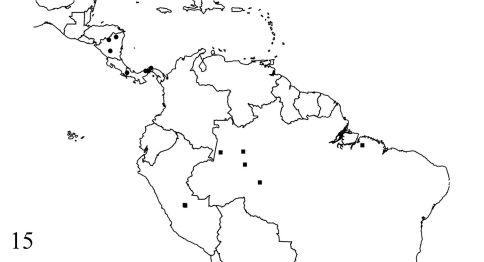
Known distribution map of Amblyodus species. Amblyodus taurus (circles) and Amblyodus castroi (squares).

The genus Amblyodus is recorded for the first time from Peru and from Brazil states of Pará and Rondônia (Fig 15). It is possible that Amblyodus castroi occurs in all the Amazonian subregion.

Characters used to diagnose the genus by Ratcliffe (2003) and Ratcliffe and Cave (2006) are confirmed with the new species described here, except for the presence of six teeth on the apex of the metatibiae. In Amblyodus castroi, that is a variable character, as the number of teeth may be five or six.

## Supplementary Material

XML Treatment for Amblyodus castroi
